# X-ray diffraction data as a source of the vibrational free-energy contribution in polymorphic systems

**DOI:** 10.1107/S2052252519003014

**Published:** 2019-05-08

**Authors:** Phillip Miguel Kofoed, Anna A. Hoser, Frederik Diness, Silvia C. Capelli, Anders Østergaard Madsen

**Affiliations:** a Department of Chemistry, University of Copenhagen, Copenhagen Denmark; b Biological and Chemical Research Centre, Department of Chemistry, University of Warsaw, Żwirki i Wigury 101, Warszawa 02-089, Poland; c ISIS Neutrons and Muons Facility, Science and Technical Facility Council, Rutherford Appleton Laboratory, Harwell Science Campus, Didcot OX11 OQX, UK; d Department of Pharmacy, University of Copenhagen, Universitetsparken 2, Copenhagen 2100, Denmark

**Keywords:** conformational polymorphs, vibrational contributions to free energy, lattice dynamical models, ADPs refinement, DFT calculations

## Abstract

In this contribution we attempt to answer a general question: can X-ray diffraction data combined with theoretical computations be a source of information about the thermodynamic properties of a given system? Newly collected sets of high-quality multi-temperature single-crystal X-ray diffraction data and complementary periodic DFT calculations of vibrational frequencies and normal mode vectors at the Γ point on the yellow and white polymorphs of di­methyl 3,6-di­chloro-2,5-di­hydroxy­terephthalate are combined using two different approaches, aiming to obtain thermodynamic properties for the two compounds. The first approach uses low-frequency normal modes extracted from multi-temperature X-ray diffraction data (normal coordinate analysis), while the other uses DFT-calculated low-frequency normal mode in the refinement of the same data (normal mode refinement). Thermodynamic data from the literature [Yang *et al.* (1989), *Acta Cryst.* B**45**, 312–323] and new periodic *ab initio* DFT supercell calculations are used as a reference point. Both approaches tested in this work capture the most essential features of the systems: the polymorphs are enantiotropically related, with the yellow form being the thermodynamically stable system at low temperature, and the white form at higher temperatures. However, the inferred phase transition temperature varies between different approaches. Thanks to the application of unconventional methods of X-ray data refinement and analysis, it was additionally found that, in the case of the yellow polymorph, anharmonicity is an important issue. By discussing contributions from low- and high-frequency modes to the vibrational entropy and enthalpy, the importance of high-frequency modes is highlighted. The analysis shows that larger anisotropic displacement parameters are not always related to the polymorph with the higher vibrational entropy contribution.

## Abstract 2

Combinations of *ab initio* calculations and modelling against X-ray diffraction data are used to derive the free energy of polymorphic crystals. The results are discussed in relation to the relative stability and phase transitions of the enantiotropically related polymorphs.

## 1. Introduction   

Nowadays polymorphism is one of the hottest topics in chemistry, physics, crystallography and the pharmaceutical industry. As polymorphs of the same substance exhibit different physicochemical properties and biological activity, it is important to gain insight about the relative stability of the given polymorphic forms. Aside from experimental studies of the relative stability of polymorphs, efforts on improving computational methods to deal with this have been of general interest to the scientific community (Marom *et al.*, 2013 ▸; Nyman & Day, 2015 ▸, 2016 ▸; Reilly & Tkatchenko, 2014 ▸).

To acquire a clear picture of the different stability of polymorphic forms – which initially have to stay close in energy for the transition from one polymorph to another to occur – it is not sufficient to evaluate the lattice energy. The atoms in a crystal lattice are not frozen, but oscillating about their average positions, hence the contribution from normal mode vibrations to enthalpy and entropy should be taken into account. It is still challenging to obtain an accurate vibrational contribution to enthalpy and entropy for molecular crystals using theoretical *ab initio* calculations, although considerable progress has been seen in recent years, in particular, the quasi-harmonic approximation seems to be a promising approach (Erba *et al.*, 2016 ▸; Heit & Beran, 2016 ▸; Ruggiero *et al.*, 2017 ▸; Brandenburg *et al.*, 2017 ▸; Červinka *et al.*, 2016 ▸). In order to improve the estimation of the vibrational contributions to enthalpy and entropy, we decided to make use of the information comprised in X-ray diffraction data. The idea is not new; attempts to estimate entropy from X-ray diffraction data analysis were first made by Cruickshank, who estimated the vibrational entropy of crystalline naphthalene on the basis of a translational-librational (Tω) rigid-body analysis (Cruickshank, 1956*a* ▸, ▸ *b*). Recently, similar approaches based on Schomaker and Trueblood’s corrections to Cruickshank’s rigid-body formalism (TLS analysis) (Schomaker & Trueblood, 1968 ▸) were used for xylitol and ribitol (Madsen & Larsen, 2007 ▸; Madsen *et al.*, 2011 ▸) and for polymorphs of 2-pyridine­carboxaldehyde hydrazine (Mazur *et al.*, 2016 ▸). Another beautiful approach which aimed at finding thermodynamic properties from X-ray diffraction data is the so-called normal coordinates analysis (NKA) (Bürgi & Capelli, 2000 ▸). This approach is more advanced than TLS analysis, allowing a more elaborate description of molecular motion, and requires multi-temperature diffraction measurements. It has been applied by Aree and Bürgi to obtain vibrational contributions for polymorphs of glycine (Aree *et al.*, 2012 ▸, 2014 ▸, 2013 ▸) and by Capelli and Bürgi to obtain the heat capacity for naphthalene (Capelli *et al.*, 2006 ▸).

A new approach we applied in this work is different from both TLS analysis and NKA modelling. In the spirit of quantum crystallography (Grabowsky *et al.*, 2017 ▸; Genoni *et al.*, 2018 ▸), we seek to enhance information that can be derived from diffraction data by relying on an ansatz based on quantum mechanics. In our previous papers (Hoser & Madsen, 2016 ▸, 2017 ▸), we introduced a normal mode refinement (NoMoRe) which enables the calculation of thermodynamic properties. In this approach, we embark from lattice dynamical models derived from periodic DFT calculations. A selected number of normal mode frequencies (ω_*j*_) are subsequently refined – *via* the calculation of Debye–Waller factors (Madsen *et al.*, 2013 ▸) – against X-ray diffraction data. The approach relies on the direct connection between the lattice dynamical model, which consists of normal mode vectors **e**(*k*|*j* **q**) and normal mode frequencies (ω_*j*_) and the atomic mean-square displacement matrices **B**_atom_(*k*) (which in turn gives the Debye–Waller factors):
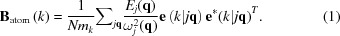
In this equation, the mean-square displacement matrix of atom *k* is obtained by the summation over all lattice modes *j* for all wavevectors **q**. *E_j_*(**q**) is the energy of mode *j* at wavevector **q**. Most of the lattice modes are high-energy intra-molecular vibrations, and these modes contribute very little to the mean square displacements – thus cannot be refined against diffraction data – and are instead kept fixed at the values obtained from DFT. Since the atomic mean square displacements correspond to mean displacements over the entire Brillouin zone, the information that we can hope to obtain from refinement of the lattice dynamical model against Bragg diffraction data are likewise mean frequencies for each normal mode. Thus, Equation (1) reduces to
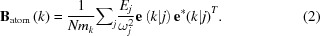
The starting lattice dynamical model is obtained from DFT calculations at the Γ point of the Brillouin zone. The normal mode vectors **e**(*k*|*j*) are kept fixed while the normal mode frequencies are refined.

In previous work we have worked at a single temperature; however, here we expand the method so that we refine one common lattice dynamical model against diffraction data from several temperatures. The temperature *T* comes in play in Equation (2) *via* the normal mode energy *E_j_*, which can be expressed as
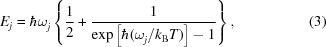
where *k*_B_ is the Boltzmann constant. Notice that several aspects of lattice dynamics are mapped into the computed harmonic vibration modes at the Γ point: phonon dispersion, anharmonic terms of the potential and thermal expansion. Therefore, the refined frequencies should be considered to represent mean frequencies over the Brillouin zone, as well as frequencies that to some extent incorporate anharmonic effects. We discuss these aspects further later in the paper. Subsequently, the obtained frequencies are used to estimate thermodynamic properties – vibrational contributions to the free energy and heat capacity.

Aside from calculations of thermodynamic properties, we are obtaining the anisotropic displacement parameters (ADPs) for all atoms, including hydrogens, which are known to be difficult to refine from X-ray diffraction data.

We name the NoMoRe procedure adapted to fit multi-temperature data mta-NoMoRe. This approach has some advantages compared with NoMoRe. The most important is that the ADPs used for such an approach can be obtained by refinement of an aspherical density model (AAM) leading to a better description than that obtained with the independent atom model (IAM).

We decided to test this new approach on the di­methyl 3,6-di­chloro-2,5-di­hydroxy­terephthalate polymorphs. Since 1800, it has been known that di­methyl-3,6-di­chloro-2,5-di­hydroxy­terephthalate can change colour very dramatically in the solid state, as well in solvent. Firstly, crystal structures of a yellow form (**Y**) and a white form (**W**) have been described by Byrn *et al.* (1972 ▸), and later on three different polymorphic forms were observed by Yang and coworkers (Richardson *et al.*, 1990 ▸; Yang *et al.*, 1989 ▸): **Y** which seems to be, thermodynamically, the most stable at low temperatures, **W** that is most stable at high temperatures; and then also a light yellow (**LY**) form which is of intermediate stability and that can be seen as a transition state (Yang *et al.*, 1989 ▸). According to previous studies (Yang *et al.*, 1989 ▸; Richardson *et al.*, 1990 ▸), polymorphs **Y** and **W** are enantiotropic, whereas **LY** is monotropic. The study by Pratik and co-workers (Pratik *et al.*, 2014 ▸) nicely illustrates a transition pathway between **Y** and **W** forms. It shows, that the **LY** form exists only on the high-energy pathway. According to our energy calculation, the **LY** form has higher energy than **W** and **Y**. Additionally, despite many attempts we were not able to crystallize the **LY** form. Hence, in this contribution we will focus only on the relative stability of **Y** and **W**. As opposed to the previous studies (Yang *et al.*, 1989 ▸; Richardson *et al.*, 1990 ▸), as well as Pratik *et al.* (2014 ▸) we are not aiming to elucidate the phase transition from **Y** to **W**, we are instead focusing on the relative thermodynamic stabilities at a given temperature by taking into account not only electronic energy, but also the vibrational enthalpy and entropy, in order to obtain the total free energy.

Both approaches, NKA and mta-NoMoRe, require multi-temperature single-crystal X-ray diffraction measurements. For **Y**, **LY** and **W** the multi-temperature data sets collected by Dunitz are available (Yang *et al.*, 1989 ▸). Unfortunately, those data were collected 30 years ago on a point detector, and the resolution achieved was only 0.8 Å. Such a resolution is not sufficient to properly deconvolute electron density and thermal motion, and to obtain high quality ADPs. Consequently, for both polymorphic forms **W** and **Y**, we present here results from new multi-temperature measurements (5, 100, 125, 150, 175, 200 K, RT), collected to a resolution of 0.65 Å. All datasets were refined with the transferable aspherical atom model (TAAM) (Dominiak *et al.*, 2007 ▸; Jarzembska & Dominiak, 2012 ▸). The dataset obtained at 5 K, where the thermal motion is reduced to its minimum, is also helpful to check the presence of disorder in the structure.

In this contribution, we use the high-quality ADPs obtained from our diffraction measurements to perform NKA and mta-NoMoRe refinements for both **Y** and **W** polymorphs. A discussion of the relevant statistical parameters obtained with the two models of both **Y** and **W** polymorphs is presented, together with an analysis of the relative contributions to the total free energy: the electronic energy from *ab initio* DFT calculations, zero point energy and vibrational contributions to enthalpy and entropy calculated from frequencies of the refined lattice dynamical models. These results are discussed in comparison with the observed phase transition temperatures, as well as with a lattice dynamical model based only on supercell periodic DFT calculations. We thereby address the overall question: can X-ray diffraction data be a source of information about vibrational contributions to enthalpy and entropy of molecular crystals?

## 2. Methods   

### 2.1. Synthesis and crystal growth   

All compounds were prepared from commercially available materials according to published procedures (Hintermann & Suzuki, 2008 ▸). The obtained products were further purified by flash chromatography on silica gel using a gradient of 0–100% ethyl­acetate in heptane. Crystals of **Y** were obtained by slow evaporation from chloro­form in NMR tubes at room temperature. Crystallization took two months. Crystallization of **W** followed a similar procedure but with ether as the solvent. The scheme below shows the synthesis of di­methyl 3,6-di­chloro-2,5-di­hydroxy­terephthalate.[Chem scheme1] 
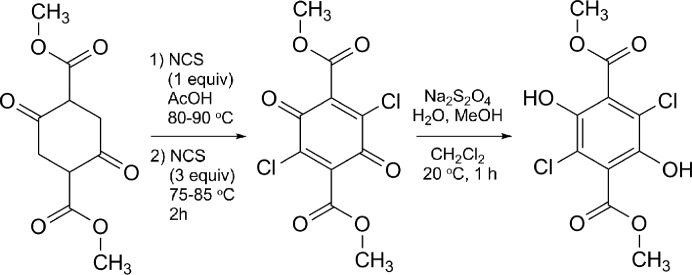


### 2.2. Data sets   

The 5 K single-crystal X-ray diffraction measurements for **Y** and **W** were carried out on a Bruker D8 Venture diffractometer using Mo *K*α radiation and helium cooling (L-He setup from Cryo Industries of America). The 100, 125, 150, 175 and 200 K single-crystal X-ray diffraction data were collected on an X-ray Agilent Technologies SuperNova diffractometer with Mo *K*α radiation. In each data collection, the temperature remained stable and within 5 K of the nominal temperatures. All data sets were collected to a resolution of at least 0.65 Å. Furthermore, to obtain more accurate ADPs, after standard IAM refinement for all measurements, a TAAM refinement was performed (Dominiak *et al.*, 2007 ▸; Jarzembska & Dominiak, 2012 ▸). Such an approach will allow a better deconvolution of the thermal motion from static charge density, and thus give more accurate ADPs than the IAM (Sanjuan-Szklarz *et al.*, 2016 ▸). TAAM refinements of positions and ADPs were carried out using *MoPro* (Jelsch *et al.*, 2005 ▸). TAAM refinements were performed in the following way: (1) structures were solved and refined by the IAM method in *OLEX* (Dolomanov *et al.*, 2009 ▸); (2) multipolar parameters were transferred from the UBDB database (Dominiak *et al.*, 2007 ▸), parameters were kept unrefined; (3) H atoms were shifted along experimental X—H directions to the values recommended by the particular database; (4) positions and ADPs for non-hydrogen atoms were refined in *MoPro* against all data. Tables containing all crystallographic details can be found in the supporting information.

### 2.3. Computational details   

Periodic *ab initio* DFT-D calculations were performed on both the polymorphs using the *CRYSTAL14* program (Dovesi *et al.*, 2014 ▸). Initial tests using the DFT functional B3LYP in combination with an empirical dispersion energy correction (Civalleri *et al.*, 2008 ▸) gave a stability order of polymorphs which was not in agreement with experimental observations, possibly due to the problems of B3LYP in describing long-range interactions (Kruse *et al.*, 2012 ▸). Therefore, we reverted to the PBE0 functional (Adamo & Barone, 1999 ▸) with a pVTZ basis set with empirically modified Grimme dispersion correction (Civalleri *et al.*, 2008 ▸). Such a level of theory was used in the calculations of total and lattice energies for polymorphs as well as for lattice dynamical calculations. Additional calculations in *CASTEP* (Clark *et al.*, 2005 ▸) confirmed the results obtained using *CRYSTAL14*, supporting the assumption that B3LYP is the culprit leading to the reversed stability order.

The starting point for the lattice dynamical calculations at the Γ point were **Y** and **W** structures obtained at 100 K, for which the atomic coordinates were optimized. For the purposes of comparison and verification of our results, we additionally conducted supercell calculations in order to derive a full *ab initio* lattice dynamical model.

Reference supercell calculations were conducted by means of DFT methods in *CRYSTAL14* in a 2 × 2 × 2 supercell, using PBE0 functional with a pVTZ basis set. In this case, we optimized both the cell parameters and atomic coordinates.

### 2.4. Normal coordinate analysis   

The multi-temperature data described above were also analysed using the NKA method by Bürgi (Bürgi & Capelli, 2000 ▸; Capelli, Förtsch & Bürgi, 2000 ▸). For both systems, **W** and **Y**, six normal modes and corresponding frequencies were refined per molecule. These modes are basically the rigid-body translations and librations in which additional internal modes, predicted to be at low frequency by DFT calculations, are allowed to mix in. In particular, two internal modes composed of the rotation of the COO group and a symmetric butterfly motion of the external groups (see Fig. S3 of the supporting information) were found to mix with a translational mode perpendicular to the plane of the aromatic ring.

Along with these temperature-dependent modes, we refined temperature-independent atomic epsilon tensors accounting for the high-frequency intramolecular vibrations. One epsilon tensor for each atom type (Cl, C, O) was refined, as well as an overall tensor accounting for any additional temperature-independent disorder in the systems. In order to describe anharmonic features associated with the thermal expansion of the crystal, a number of mode-specific Grüneisen parameters were included in the fitting procedure. An overall inertial coordinate system with the *x* axis along the long axis of the molecule, the *y* axis orthogonal to it in the plane of the benzene ring and a *z* axis to complete a right-handed Cartesian set, was used for all calculations. The inertial system for the epsilon tensors was defined for each atom with the main bond in the *x* direction, the *y* direction tangential to it in the plane of the benzene ring and the *z* direction to form a right-handed coordinate system. The refinement statistics and details of the models of motion can be found in the supporting information.

### 2.5. Normal modes fitting to multi-temperature ADPs   

The *NoMoRe* program (Hoser & Madsen, 2016 ▸, 2017 ▸) was adapted to refine against multi-temperature ADPs. The starting ansatz is a lattice dynamical model derived at the Γ point using periodic DFT calculations. The frequency of low-energy normal modes are then updated iteratively using the method of least squares in order to minimize the difference between the computed ADPs and the experimentally derived multi-temperature ADPs. Throughout the refinements, the normal mode coordinates are retained from the *ab initio* lattice dynamical model. Because the model is derived at the Γ point only, the three acoustic modes are assigned initial frequencies of 50 cm^−1^.

In mta-NoMoRe, similarly as in the case of NoMoRe, not all frequencies from DFT computations are fitted, but only the frequencies of acoustic and low-energy optical phonons. There are several reasons for such an approach: first of all, the highest contribution to mean square displacements comes from the acoustic modes and soft, low-energy optical modes. The acoustic modes at the Γ point are zero, but because the ADPs correspond to the mean square displacements as a mean value over the Brillouin zone, these modes give the largest contributions to the atomic mean square displacements. As a consequence, we will obtain an estimation of the mean value of acoustic mode frequencies.

Secondly, the high-frequency modes corresponding to internal molecular vibrations are accurately described by DFT calculations at the Γ point and hence there is no need to refine them. Unfortunately, there are only few small, rigid systems [like naphthalene (Natkaniec *et al.*, 1980 ▸; Capelli *et al.*, 2006 ▸)] for which the boundary between external and internal modes is well defined. Therefore, it is not straightforward to decide which modes one should refine. As a result, the final refined model will depend on the quality of the measurements, the quality of theoretical computations as well as on the number of refined frequencies. In the case of studied polymorphs another difficulty arises from the fact that **Y** and **W** polymorphs differ by the number of molecules in the asymmetric unit. As a consequence, after the calculations we obtain twice as many modes and related frequencies (78 and 156 for **Y** and **W**, respectively).

Thus, herein we present and critically discuss two different mta-NoMoRe models.

(*a*) AO – *i.e.* ‘acoustic only’, meaning that only the frequencies of the acoustic modes are refined

(*b*) FG – *i.e.* ‘frequency gap’ model, meaning that vibrational data have been analysed for a gap between low- and high-frequency modes, and only the frequencies below the gap have been refined. In our case, we have refined 17 and 26 modes for the yellow (**Y**) and white (**W**) polymorphs, respectively. In both cases, the frequencies of these modes were below *ca* 200 cm^−1^.

Both AO and FG models were refined against the ADPs corresponding to the measurements at 100, 125, 150, 175 and 200 K. As we used only non-hydrogen atoms during refinement, and we have *U_ij_* parameters from five temperatures, the models were refined against 240 and 480 *U_ij_* parameters for **Y** and **W**, respectively.

In order to judge the quality of the fits against experimental X-ray ADPs, the following residuals were evaluated:
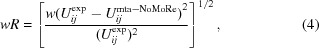
where ***w*** stands for 1/σ^2^, *U_ij_* ^exp^ are experimental *U_ij_*, and 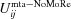 are *U_ij_* obtained after mta-NoMoRe, and
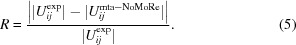



### 2.6. Evaluation of thermodynamic functions   

All thermodynamic functions were calculated using known statistical thermodynamics equations. For a given compound the free energy (*G*) is calculated as shown in the following equations:
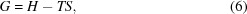
where *H* stands for enthalpy, *T* is the temperature in K and *S* is the entropy. The enthalpic part can be divided into *U*, the crystal packing energy, and *p*ΔV, pressure × volume, terms which take into account the expansion of the unit cell with temperature
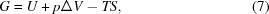
where *p*Δ*V* is calculated at a pressure of 1 atm, whereas
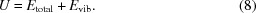

*E*_total_ is an electronic energy, accessible from DFT calculations and the contribution from vibrational energy, *F*_vib_, is equal to
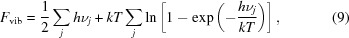
where the summation runs over frequencies ν, *h* is Planck’s constant and *k* is the Boltzmann constant. The first part of the equation is the zero-point energy (*ZPE*), whereas the second part is the contribution to enthalpy (*H*_vib_) from normal mode vibrations.
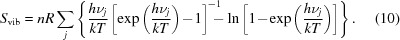
In the case of mta-NoMoRe, all the frequencies obtained from the calculations in *CRYSTAL* and later refined were used in order to obtain full vibrational contributions to free energy. In the case of NKA, the first four frequencies for **Y** and eight for **W** from the periodic DFT calculations at the Γ point were replaced with frequencies from the NKA analysis and then such set of frequencies was used for further estimation of the vibrational entropy and enthalpy.

## 3. Results and discussion: structures from X-ray data   

### 3.1. Structures of **Y** and **W** polymorphs   

**Figure 1:**
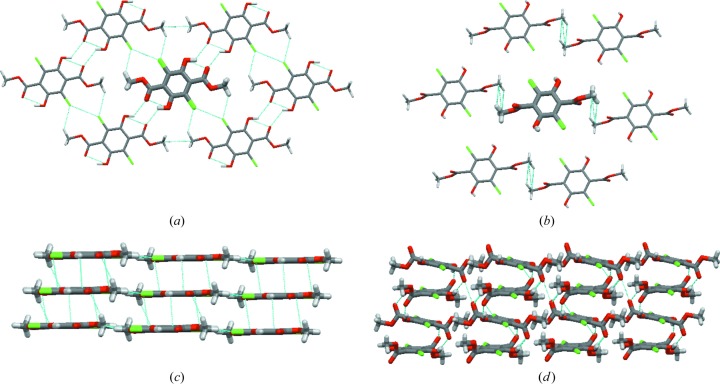
Structural features of **Y** [(*a*), (*c*)] and **W** [(*b*), (*d*)].

The crystal structures of the two polymorphs of interest, **Y** and **W**, have already been described previously (Yang *et al.*, 1989 ▸), so here we will just briefly mention the structural differences between them (Fig. 1 ▸). **Y** and **W** are conformational polymorphs. In the case of **Y** the meth­oxy­carbonyl group lies in the same plane as the benzene ring. Such an orientation of the meth­oxy­carbonyl groups enables for intramolecular hydrogen bond formation with the hydroxyl group. In the case of **W**, the meth­oxy­carbonyl group is rotated and almost perpendicular to the benzene ring, and the hydroxyl group is involved in intermolecular hydrogen bonding. As a consequence, two different types of layered structures are formed: in the case of **Y** hydrogen bonds are formed within the layers, and the layers are stabilized by π⋯π stacking interactions [Figs. 1 ▸(*a*) and 1 ▸(*c*)], whereas in the case of **W**, hydro­gen bonds are formed between the layers [Figs. 1 ▸(*b*) and 1 ▸(*d*)].

Due to the existence of intermolecular hydrogen bonding between layers, **W** could be expected to have a higher density than **Y**. Nevertheless, it is the **Y** polymorph which has a slightly higher density and packing coefficient than **W** (at 5 K: 1.823 g cm^−3^ versus 1.776 g cm^−3^, 0.76 *versus* 0.75; at 298 K: 1.752 g cm^−3^ *versus* 1.704 g cm^−3^, 0.73 versus 0.73).

**Figure 2:**
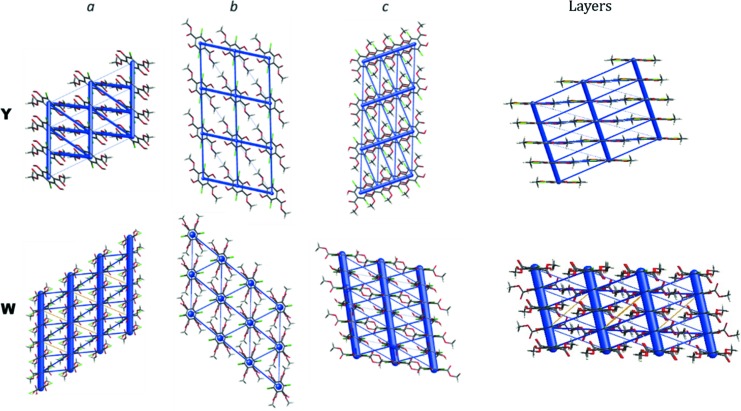
Energy frameworks generated for **Y** and **W** polymorphs. Total energy along *a*, *b* and *c* directions.

Differences in crystal density of **Y** and **W** can be explained on the basis of the so-called energy frameworks (Turner *et al.*, 2015 ▸) (see Fig. 2 ▸). By analysing the strongest contributions to the total energy between layers, which are formed in both structures, it can be observed that in the case of **W** there are very strong interactions between molecules, which are forming columns (*ca* −100 kJ mol^−1^, so high due to hydrogen bonding), but interactions between columns and within each layer are very weak (−14 and 16 kJ mol^−1^). In the case of the **Y** polymorph, interactions between layers (−47 and −32 kJ mol^−1^) and within a layer (−23 and −16 kJ mol^−1^) are more similar in energy. Thus, we may conclude, that in **Y** the interactions are more isotropically distributed in three directions than in the case of **W**, and this might explain its higher density. The differences in intermolecular interactions are also reflected in the anisotropy of the thermal expansion, which is much larger for **W** than for **Y**, as witnessed by plots of the expansivity indicatrices given in Fig. S1 of the supporting information.

### 3.2. ADPs from multi-temperature measurements   

**Figure 3:**
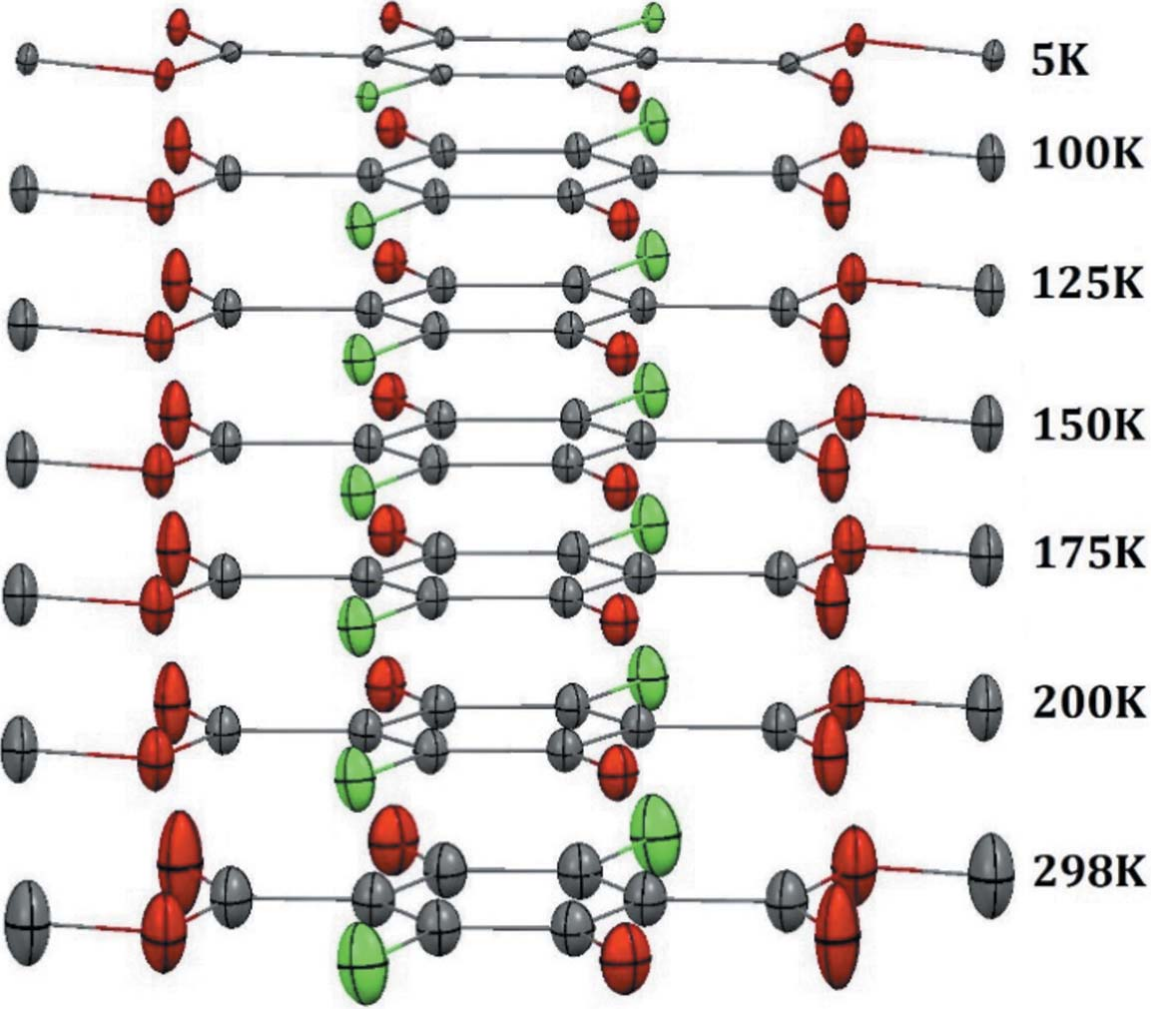
ADPs (50% probability level) at different temperatures for the **Y** polymorph. Hydrogen atoms have been omitted for clarity.

Thermal ellipsoids for the different temperatures for **Y** and **W** are presented in Figs. 3 ▸ and 4 ▸. For both systems, we observe – as expected – a gradual increase of ADPs as a function of temperature. The high-quality 5 K measurements demonstrate that at this low temperature there is no static disorder, and confirm that there are no phase transformations in the temperature range 5–100 K.

Comparing the ADPs for **W** and **Y**, we observe much larger ADPs at room temperature for atoms in the meth­oxy­carbonyl group of **Y**. The same trend was observed by Yang *et al.* We also observe a significant difference in the case of the chlorine atoms. On the other hand, the ADPs for the benzene ring are comparable for both polymorphs (within three standard uncertainties).

Given the gradual increase of the ADPs with temperature, it is reasonable to interpret this increase in ADPs of **Y** compared with **W** as due to lower energy of equivalent low-frequency lattice vibrations. Yang *et al.* (1989 ▸) demonstrates that the **Y** system can be understood as a vibrating rigid body with a specific non-rigid unit, *i.e.* a libration of the meth­oxy­carbonyl group, which temperature dependence is different from the translational and librational motion of the molecule. They propose that this motion is related to the phase transition mechanism.

### 3.3. Anharmonic motion or disorder?   

**Figure 5:**
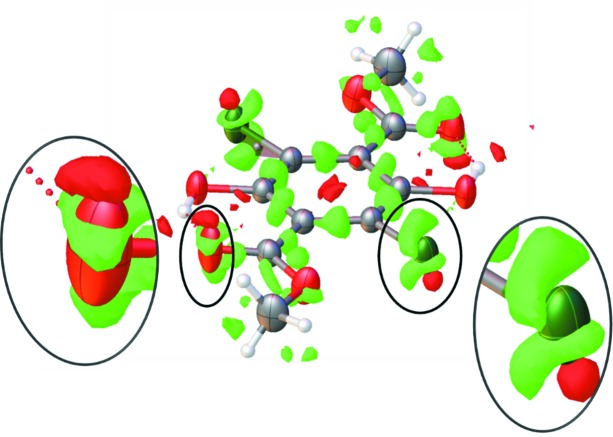
Residual density in the vicinity of the meth­oxy­carbonyl group and chlorine yellow polymorph before TAAM refinement, isovalue level: 0.1 e Å^−1^, green = unmodeled density.

The smooth increase of ADPs as a function of temperature, which is observed for both polymorphs, in combination with the featureless residual density maps of the TAAM refinements would seem to rule out the possibility of disorder as an explanation for the large differences in ADPs between **Y** and **W**. However, to our surprise, the room-temperature residual electron density of the **Y** polymorphs shows clear signs of disorder or anharmonicity. Despite the elongated ADPs of the meth­oxy­carbonyl group and chlorine, there are still distinct residual density peaks near the Cl and O atoms, which exhibit the characteristic ‘shashlik’-like pattern (Meindl *et al.*, 2010 ▸; Herbst-Irmer *et al.*, 2013 ▸) for anharmonic motion (see Fig. 5 ▸). This feature is not coupled with any sign of diffuse scattering (at least not detectable by our CCD detector) which would indicate long-range correlation of disorder.

Although those residual density features are not visible for models obtained below room temperature, we hypothesize that they probably are also present to some extent at lower temperatures, giving rise to some of the elongation of ellipsoids of **Y** which is not seen in **W**. As will be evident below, this might be the reason for higher residuals associated with the mta-NoMoRe modelling of **Y** as opposed to **W**.

By analyzing the lattice dynamical models obtained from DFT calculations we found one optical low-frequency mode which corresponds to an out-of-plane libration that could give rise to the large displacements of the meth­oxy­carbonyl and chlorine atoms in **Y**. Scanning the potential energy surface along the normal mode coordinate (see Fig. S4), the mode shows signs of anharmonicity, but the magnitude of the deviations from the harmonic case are not large enough to explain the experimentally observed elongation of ellipsoids and the residual densities. Obviously, also other vibrational modes give contributions to the mean square displacement of the meth­oxy­carbonyl group, which we did not scan along normal mode vectors. Comparison of lattice dynamical models obtained from Γ-point calculations using fixed cell parameters at 100 and 300 K reveals that for this particular normal mode the frequency is changing from 47 to 40 cm^−1^, which supports the hypothesis that this low frequency mode is strongly affected by anharmonicity.

## 4. Results and discussion: thermal motion analysis   

### 4.1. TLS and multi-temperature normal coordinate analysis   

In the work by Dunitz and co-workers, the displacement parameters are analysed using a TLS model including attached rigid groups in order to describe the additional intramolecular degrees of freedom, namely the rotation of the meth­oxy­carbonyl group. We repeated this analysis on our new TAAM models refined against high-resolution data. First of all, we observe improved fits of the model against the low-temperature data. We ascribe this to the better deconvolution of the thermal motion from the static charge densities due to the use of the TAAM approach. In terms of the amplitudes of vibration (or frequencies), the results are within one standard deviation for **W**. For **Y**, we obtain somewhat different values for the librational modes (at room temperature, we obtain 38, 50 and 55 cm^−1^, whereas Dunitz and coworkers obtained 32, 50 and 68 cm^−1^). The amplitude of motion of the meth­oxy­carbonyl group agrees also to within one standard uncertainty. It is interesting to note that the TLS model including an attached rigid group is in very good agreement with the **Y** data (the *R*value is 3% for all temperatures).

It should be noted here that in Dunitz and coworkers analysis of ADPs, a model of motion is used at each temperature to fit the experimental data. In order to go beyond the standard TLS-type analysis, the NKA approach uses a unique set of normal mode frequencies and eigenvectors to describe the diffraction data at all measured temperatures. In addition, it allows us to separate the temperature-independent contribution to ADPs coming from high-frequency normal modes and refine them as additive tensorial quantities for each atom type or group of atoms.

In the NKA analysis of the X-ray diffraction data for **Y** and **W**, a simple model consisting of the six rigid-body motions and three epsilon tensors to describe the temperature-independent contributions would better fit the **W** than the **Y** data, but in both cases would only describe about 87% of the ADPs at all temperatures. A more complex model, including internal modes mixing in with the lattice modes and Grüneisen-type parameters to account for anharmonicity was necessary in order to account for 93% of the total ADPs at all temperatures. The translational frequencies obtained are around 32–51 cm^−1^ and the librational frequencies are between 45 and 80 cm^−1^, in agreement with those of Dunitz. We note in particular that the highest librational frequencies, *i.e.* those requiring higher energies, are those mostly affected by anharmonicity and are different not only in the two polymorphs, but also between the two molecules in the asymmetric unit of the **W** polymorph. In the case of the **Y** form, the largest frequency (76 (7) cm^−1^) corresponds to a mix of the L_*x*_ (45%) and L_*y*_ (50%) librations, *i.e.* a motion that disrupt the in-plane hydrogen bonding pattern. For the **W** polymorph, the situation is different for the two molecules in the asymmetric unit but in both cases involving the L_*x*_ libration, which affects the inter-layer hydrogen bonding pattern. We further note that four out of the six refined frequencies for **Y** are strongly affected by anharmonicity and the corresponding refined Grüneisen parameters fall in the range 5 (1) to 7.0 (1). In the case of the **W** polymorph, only two frequencies are affected by anharmonicity, in particular the T_*z*_ translation that mixes in significantly with the rotation of the COO group and the butterfly deformation. Overall the values of the refined Grüneisen parameters are in the range 4.7 (8) to 7.1 (6), with the largest values about twice as large as those reported for glycine (Aree *et al.*, 2012 ▸, 2014 ▸, 2013 ▸) and HMT (Bürgi, Capelli & Birkedal, 2000 ▸).

### 4.2. Analysis of ADPs from mta-NoMoRe   

**Figure 6:**
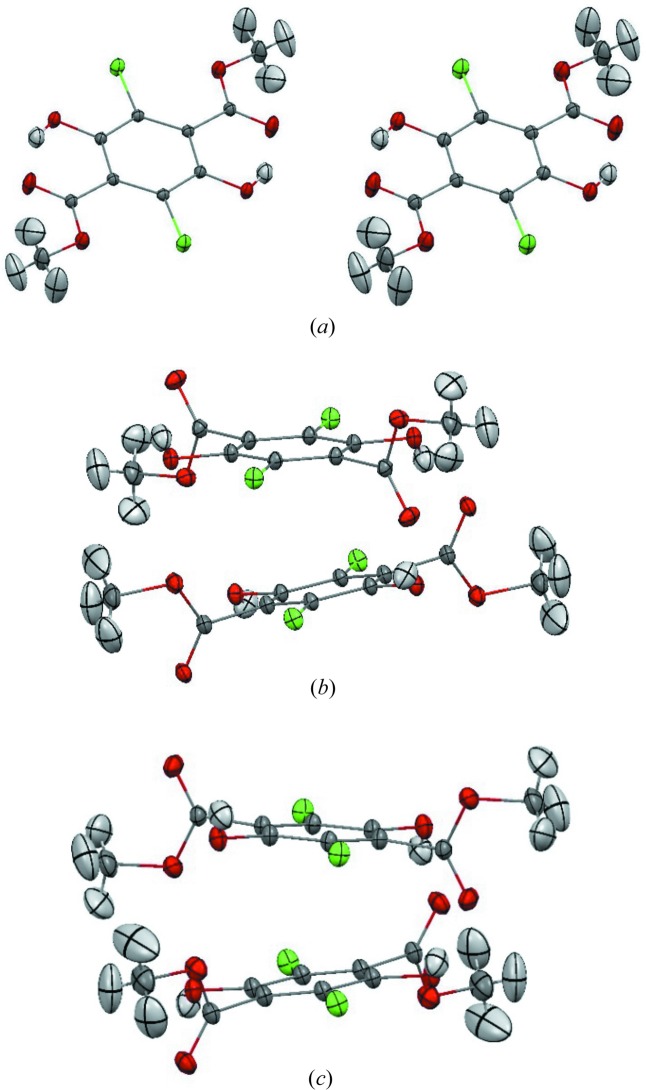
ADPs calculated from frequencies after mta-NoMoRe for **Y** at (*a*) 150 K and (*b*) 200 K and for **W** at (*c*) 150 K and (*d*) 200 K for the FG model.

From the mta-NoMoRe model we can derive ADPs at different temperatures. In Fig. 6 ▸ we present the thermal ellipsoids obtained from our model at temperatures from 100 K to 200 K. Visually the ADPs appear similar to the results typically obtained from X-ray TAAM refinements. The ADPs for the hydrogen atoms in the methyl groups have size and direction which correspond to that usually observed in models based on neutron diffraction data.

Refinement statistics (*wR*2 and *R*2, Table 1 ▸) and tensorial plots Δ*U*_*ij*_ (so-called *PEANUT* plots, see Fig. S5) reveals that the mta-NoMoRe model for **Y** is in worse agreement with the experimental ADPs than the model for **W** due to difficulties in describing the simultaneous motion of the meth­oxy­carbonyl group and the aromatic ring.

### 4.3. Frequencies obtained after application of different models of mta-NoMoRe   

**Figure 7:**
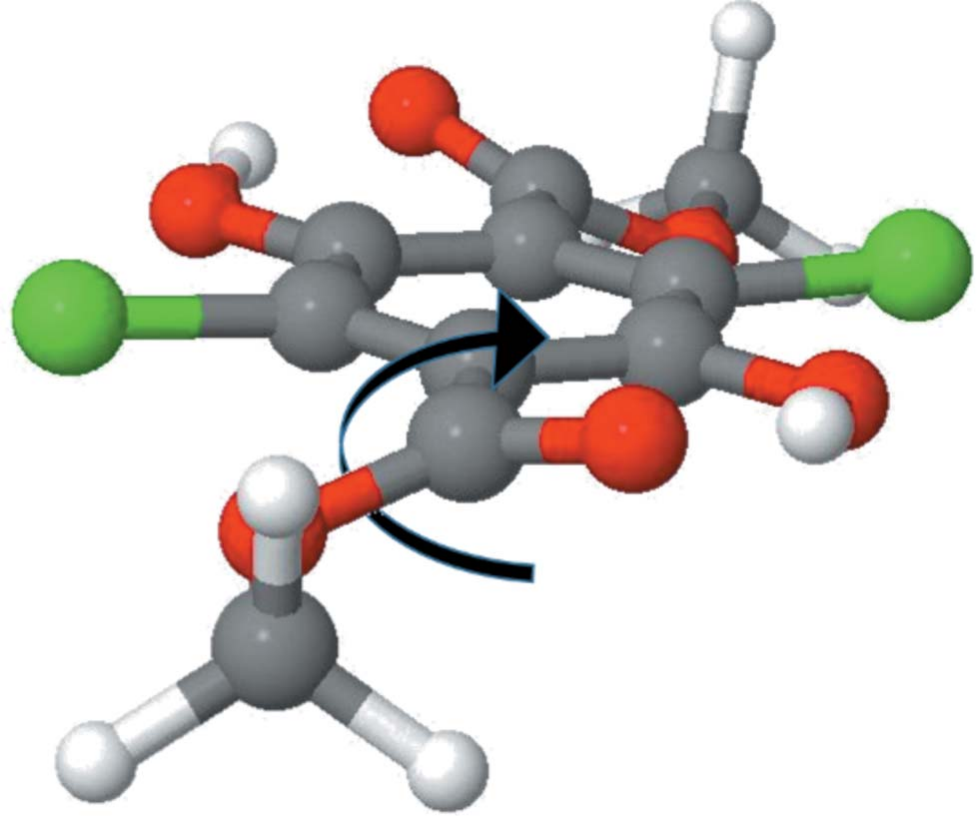
Internal mode with low frequency that is present in the **Y** polymorph.

One of the key advantages of mta-NoMoRe is that we are obtaining estimates of frequencies for acoustic modes, which are difficult to obtain accurately from calculations. In the case of **W**, those estimates are stable to changes in our model, such as the refinement of low-frequency optical modes. For **Y** the refinement is more complicated. The refinement of optical modes give strong correlations between the optical and acoustic modes, causing large changes in the acoustic mode frequencies. The large-amplitude motion of the meth­oxy­carbonyl group about the C—C bond (see Fig. 7 ▸) is primarily projected onto a low-frequency optical mode which corresponds purely to internal motion. The DFT estimate of the frequency is 77 cm^−1^, and upon refinement the frequency becomes even lower (45 cm^−1^). The residual density features (Fig. 5 ▸) and the fact that the arc-motion of the metoxycarbonyl group is described by ADPs (ellipsoids) adds to the difficulties in modelling **Y**. In contrast, the **W** metoxycarbonyl oxygen atoms are hydrogen bonded, leading to a smaller amplitude of motion and thus easier modelling. Moreover, the models from NKA clearly suggests that the motion of the meth­oxy­carbonyl group in **Y** is coupled with the ‘butterfly motion’ that preserves the intramolecular hydrogen bonding and creates the ‘arc motion’ effect.

### 4.4. Comparison of mta-NoMoRe frequencies for acoustic modes with estimates of acoustic mode frequencies from supercell calculations   

In the case of the **W** polymorph, frequencies obtained after mta-NoMoRe for acoustic modes are in good agreement with estimates of acoustic modes frequencies from supercell calculations, and they remain the same while the number of refined modes is increasing. For the **Y** polymorph, when we are refining only acoustic modes, we are obtaining frequencies similar to those from supercell calculations, but when we are increasing the number of refined modes, the frequency of mode number one is increases dramatically (see FG, Table 2 ▸), whereas the frequency of mode number four decreases. Careful visual inspection of these modes reveals that the motion of mode number one is strongly correlated with that of mode number four.

## 5. Results and discussion: relative stability of **Y** and **W**   

According to DSC measurements (Yang *et al.*, 1989 ▸; Richardson *et al.*, 1990 ▸), the **Y** form is thermodynamically stable at low temperature, and somewhere between 375 and 415 K the phase transition to the **W** form occurs. The DSC measurements indicate an endothermic process with a enthalpy change of about 1.5 kJ mol^−1^. Furthermore, Dunitz and co-workers state that the crossover in the **Y**/**W** free-energy curves must be between room temperature and 343 K according to the observations made with the video-microscope (Richardson *et al.*, 1990 ▸).

In this paper we will discuss two different approaches that make use of structures obtained from single-crystal X-ray diffraction data to get relative stability of polymorphs at different temperature ranges.

### 5.1. Stabilities based on energies at geometries from X-ray diffraction   

**Figure 8:**
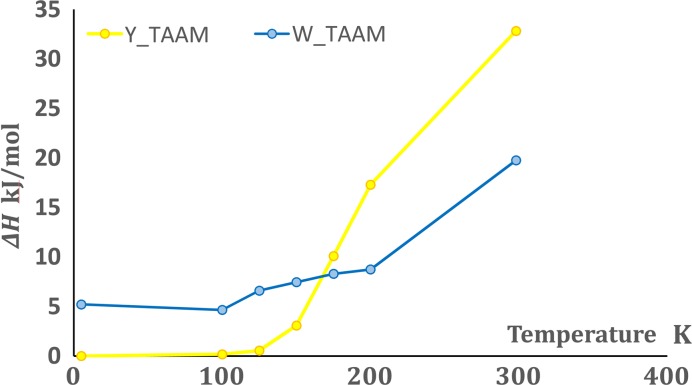
Single-point calculation results: differences in electronic energy computed for structures obtained from single-crystal X-ray diffraction measurements at different temperatures for both polymorphic forms.

The first approach is rather simple and relies solely on atomic coordinates and cell parameters obtained by X-ray diffraction at a range of temperatures. At each temperature, we have computed the DFT-D energy by single-point calculations without optimizing cell parameters and atomic coordinates, but only elongating X—H bonds to standard neutron values. The influence of temperature is taken into account simply by differences in cell parameters and atomic coordinates as obtained from X-ray experiments. Interestingly, even by using this basic approach, we see that the **Y** form has lower energy than **W** at low temperatures, whereas somewhere about 175 K there is a changeover and the **W** form exhibits lower energy (see Fig. 8 ▸). Therefore, **Y** is more stable at low temperatures, whereas **W** is stable at high temperatures. We observe some small differences between energies obtained after calculations on geometries obtained by us and those obtained 30 years ago by Dunitz, but the trend is the same. The magnitudes of differences in energies between **Y** and **W** are quite large, probably because we used the experimental structures with a fairly crude estimate of the H-atom positions. To test this hypothesis, we fixed the cell parameters for the 100 K and room-temperature structures and performed a geometry optimization of the atomic coordinates. The results show the same trends, but with much smaller energy differences: **Y** is more stable by 3 kJ mol^−1^ at 100 K, and least stable by 0.8 kJ mol^−1^ at room temperature. It is evident that the change in electronic energy caused by thermal expansion plays a large role for the change in relative stability of these polymorphs, as also indicated in a recent study (Heit & Beran, 2016 ▸). In this simple model, the temperature of the phase transition is lower than expected, and moreover, we are not taking into account contributions to the free energy from vibrations explicitly. Furthermore, this approach indicates an enthalpy – rather than entropy – driven phase transition (Kocherbitov, 2013 ▸) which is not in agreement with the DSC measurements.

### 5.2. Stabilities based on free energies   

In our second approach, aside from the total electronic energy, we are considering vibrational contributions to enthalpy, entropy and zero-point energy. These contributions are derived from either purely *ab initio* calculations or from the NKA or mta-NoMoRe approach (see *Methods* [Sec sec2] for details). In Table 3 ▸, we summarize the contributions to the differences in free energy of **Y** and **W** at 300 K using six different approaches. Below we will discuss the results.

#### 5.2.1. Electronic energy   

Theoretical single-point *ab initio* DFT calculations of the total energies of **Y** and **W** 100 K structures show that for **Y** the energy is lower by 4.49 kJ mol^−1^. Relaxation of coordinates changes the difference to 3.26 kJ mol^−1^ and full geometry optimization of both cell parameters and coordinates leads to 4.30 kJ mol^−1^.^1^ These values are in the range of differences which we would expect for conformational polymorphs (Cruz-Cabeza *et al.*, 2015 ▸). In the case of the mta-NoMoRe approaches, we were considering what to include in the free-energy calculations: either the electronic energy obtained for Γ-point calculations after geometry optimization, or rather the energies obtained from single-point energy calculations at different temperatures. As we have seen above, the change in electronic energy due to thermal expansion gives a contribution to the change in polymorph stabilities (Fig. 8 ▸). In the following models, we include vibrational contributions to obtain total free energies, and we choose to use energies derived from coordinate-optimized structures (100 K) which are consistent with the approach we use for deriving lattice dynamical models.

#### 5.2.2. Vibrational contributions: *ab initio* Γ-point calculations   

The first approach takes the vibrational contribution from the Γ point only, and corresponds to our starting ansatz for the NoMoRe refinements. Of course, the thermodynamics from this model are deficient because the acoustic modes have zero frequency at the Γ point and are not considered. Clearly the vibrational contributions obtained only at the Γ point differ significantly from the results obtained from supercell calculations, and the differences arise mostly from differences between supercell and Γ-point calculations for the **Y** polymorph (see Table S4 of the supporting information). The contribution from the acoustic modes plays a more significant role for the **Y** polymorph because of the smaller number of optical phonons. In this way, the number of atoms in the unit cell becomes an important aspect in understanding the differences in thermodynamic properties between these polymorphs. At room temperature this model estimates **W** to be the most stable, but the thermodynamic phase transition temperature is below 100 K, much too low compared with experimental observations.

The frequencies at the Γ point are generally slightly larger than the mean values over the full Brillouin zone, and of course there is no contribution from acoustic mode frequencies. This means that a pure Γ model will have too small contributions from entropy and enthalpy.

#### 5.2.3. Vibrational contributions: *ab initio* supercell calculations   

This model is close to the approach one could take in a prediction of crystal structures. The experimental unit cell and coordinates were optimized to obtain the minimum electronic energy, and the vibrational contributions were obtained by summing the frequencies of points in the Brillouin zone compatible with a 2 × 2 × 2 supercell. This is a purely harmonic approach and does not consider thermal expansion. It estimates the temperature of the phase transition to be around 300 K, very close to that observed experimentally. As opposed to the Γ-point calculations, the supercell ones take the acoustic modes into account, giving rise to a dramatic change of the entropic factor Δ*ST*_(**Y**−**W**/2)_ of almost 8 kJ mol^−1^. This is mostly due to huge changes in the entropy of **Y** (see Table S4).

In general, from our observations we see that the less atoms the system has, the more important the acoustic modes are, and thereby their contribution to the free energy.

Differences in *ZPE* obtained from this model seem to be surprisingly high, around 2.7 kJ mol^−1^. According to Nyman & Day, this difference between polymorphs is usually lower than 0.7 kJ mol^−1^ (Nyman & Day, 2015 ▸). However, it is worth noting that in our calculations we are using a different approach to than of Nyman & Day (2015 ▸): their study was done using force fields while in our study we use DFT methods. Our results are in better agreement with other estimates based on DFT which confirm the importance of *ZPE* contribution to free energy when the relative stability of polymorphs is investigated (Rivera *et al.*, 2008 ▸).

Despite the very convincing results of this model, we should remember that it is a purely harmonic model. We know that the systems – especially the **Y** polymorph – show strong anharmonic features, as showed by the elongated ellipsoids and residual density of the Cl and meth­oxy­carbonyl groups. We are probably experiencing a fortuitous cancellation of errors in this model. It is likely that in an anharmonic model the thermal expansion would lead to lower vibrational frequencies and consequently increased entropies, but this effect would be counteracted by changes in the lattice energies due to larger intermolecular distances.

#### 5.2.4. Vibrational contributions from NKA   

One way of incorporating the effect of anharmonicity is the use of the NKA. In order to study the effect of anharmonicity on the free energies, we report in Table 3 ▸ two different results for the NKA models. The first model uses the frequencies without the Grüneisen-correction, *i.e.* neglecting the thermal expansion. The second incorporates the effect of anharmonicity. In both cases we calculate the thermodynamic functions at 300 K. For these calculations we combined the NKA frequencies with the high-frequency modes obtained at the Γ point from periodic DFT calculations.^2^

The thermodynamic properties obtained from NKA frequencies augmented with intramolecular frequencies from DFT calculations are in good agreement with results from *ab initio* supercell calculations. The best agreement is found when the NKA model without anharmonicity is used. This is reasonable because the supercell calculations are not taking into account anharmonicity.

It is evident from Table 3 ▸ that the change in the entropic contribution is the one most affecting the final changes in Δ*G*. We can assume that anharmonicity plays a major role in this term and therefore in stabilizing the **Y** form over the **W** one. The overall result that **W** becomes more stable at higher temperatures is retained; however, the phase transition temperature becomes higher throughout the six models.

#### 5.2.5. Vibrational contributions: mta-NoMoRe_AO model   

As our estimates of acoustic mode frequencies obtained by fitting only acoustic modes to the ADPs are in good agreement with results obtained after supercell calculations, also, vibrational contributions to *ΔE*_(**Y**−**W**/2)_ obtained from the AO model start to become much closer to results obtained from supercell calculations. However, vibrational contributions obtained from this model are not the same as from supercell calculations: differences arise from high-frequency modes. This model correctly predicts that **W** is stabilized by entropy at higher temperatures, however, the phase transition temperature is overestimated – it is around 600 K. This model does not explicitly take into account anharmonicity, so the thermodynamic properties are slightly different than the anharmonic NKA model described above. Possibly some of the anharmonic features which are reflected in the temperature evolution of the ADPs are incorporated into this model in terms of lowering frequencies.

#### 5.2.6. Vibrational contributions: mta-NoMoRe_FG model   

Surprisingly, fitting a larger number of modes leads to an excessive entropic contribution in the case of the yellow polymorph (see FG model, Table 3 ▸), which might be caused by strong mixing of low- and high-frequency modes. Additionally, it is worth remembering that for **Y** the final fit of frequencies to ADPs wasn’t perfect due to possible disorder or anharmonicity. Some frequencies, which were forced to describe too large experimental ADPs, might become too small and lead to too high entropy. As not all ADPs are affected by disorder or anharmonicity, it was not an issue in the case of the mta-NoMoRe_AO model.

#### 5.2.7. Free energy and the phase transition temperature   

The model that comes closest to the experimental phase transition temperature is the combination of intramolecular frequencies from theoretical DFT calculations and frequencies from NKA corrected for anharmonicity (with Gruneisen parameter). Purely theoretical approaches such as Γ-point and supercell DFT calculations underestimate the phase transition temperature, whereas both mta-NoMoRe models overestimate it. None of these approaches take into account anharmonicity. Moreover, it is important to notice that in mta-NoMoRe and NKA we used an electronic energy obtained after geometry optimization of coordinates in the experimental unit cell. A change in electronic energy of around 1 kJ mol^−1^ (from *ca* −3 to −2 kJ mol^−1^) will result in lowering of the phase transition temperatures by about 150 K. Therefore, it is important to obtain an accurate electronic energy and to consider to what extent the phase transition temperatures are sensitive to changes in energy.

## 6. Considerations on the use of ADPs to derive vibrational enthalpy and entropy   

At the end of their first paper on di­methyl-3,6-di­chloro-2,5-di­hydroxy­terephthalate, Yang *et al.* (1989 ▸) asked the question: ‘*Why has our intuition led us astray in expecting that the crystal with the larger atomic ADPs should have the greater entropy? … The problem is left to the theoreticians.*’ Although we are not theoreticians, but crystallographers, we will try to answer their question.

First of all, it is worth recalling that ADPs are a dustbin for all experimental errors. Incorrect absorption correction, incomplete data, small amounts of disorder, diffuse scattering and thermal diffuse scattering, twining, problems with deconvolution of thermal motion, and charge density all can and will dramatically affect the ADPs.

**Figure 9:**
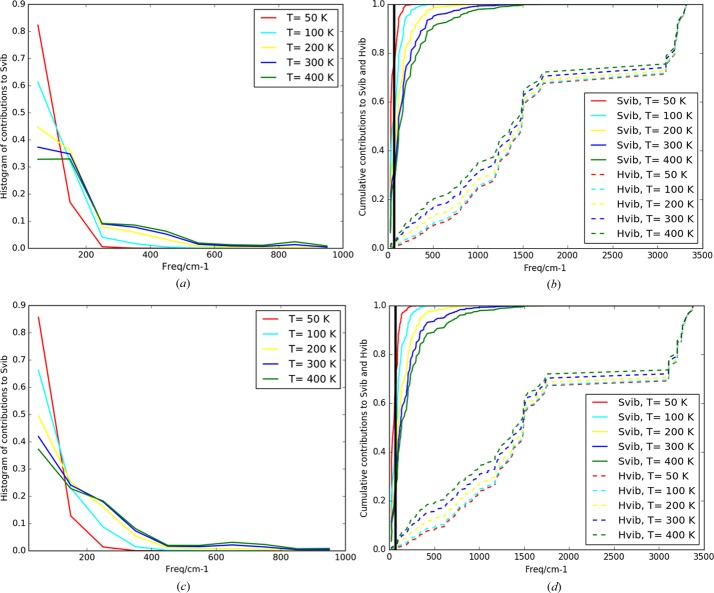
Histogram of percentage contribution of given frequencies to vibrational entropies for (*a*) **Y** and (*c*) **W**. Bin size = 100 cm^−1^. Cumulative contributions of a given frequency to vibrational entropy and enthalpy for (*b*) **Y** and (*d*) **W**.

But even assuming that the ADPs are perfect, it is not straightforward that the compound with larger ADPs will have the higher entropy, especially in the case of conformational polymorphs. For the **W** polymorph, there is an important contribution to entropy that arises from high-frequency modes, and it is becoming increasingly important (see Fig. 9 ▸) as we approach the phase transition temperature. Therefore, we conclude that in the case of conformational polymorphs it is not generally true that differences between vibrational entropies are related only to the differences in acoustic and low-energy optical modes. Moreover, even when the conformations of molecules are similar, one should take into account the number of molecules in the asymmetric unit.

## 7. Conclusions   

In this work, we have investigated a range of approaches to obtain the relative thermodynamic stabilities of a pair of polymorphic molecular crystals. All approaches capture the most essential feature of the systems: that the polymorphs are enantiotropically related, with the **Y** form being the thermodynamically stable system at low temperature, and **W** at higher temperatures.

A reasonable estimate of the phase transition temperature was obtained using a purely *ab initio* supercell calculation. Unfortunately, such calculations have high computational cost. Furthermore, they do not include anharmonicity, as reflected in the thermal expansion of the crystals.

When the effect of anharmonicity is incorporated into the model by refinement against multi-temperature ADPs, as was the case with the NKA model, we obtained the best agreement of phase transition temperatures with experimental observations. Additionally, we observe that different frequencies are affected by anharmonicity in the case of both polymorphs, and thus we obtain a change in the relative stabilities. Our results indicate that anharmonic features may change the solid-state phase transition temperature substantially.

We have demonstrated in this work that an analysis of Debye–Waller factors from crystallographic models obtained at multiple temperatures using a quantum-mechanical lattice dynamical ansatz is sufficient to give the essential thermodynamic differences between the two polymorphs on a qualitative level. In the case of the **W** polymorph, we obtain a good fit to the experimental data, whereas for **Y** the differences can most likely be explained as anharmonic features. For both polymorphs, it seems possible to extract valuable information on the mean frequencies of the acoustic modes. These are, from a computational point of view, the most difficult vibrations to assess because they reflect the curvature of the intermolecular potential energy surface. As an important bonus, we obtain a physically plausible description of the H ADPs.

In the lattice dynamical model, we only refine the frequencies of a selected number of lattice vibrations. The normal mode vectors are retained from the quantum-mechanical ansatz. This means that the flexibility of our model is rather limited. In the case of the **Y** polymorph, the differences between the model and the experimental ADPs triggered further scrutiny of the experimental data, revealing that anharmonicity is an important aspect of the dynamics of **Y**.

It is quite common opinion that large ADPs are associated with high vibrational entropy. This is not always true, as ADPs are a dustbin for all experimental problems. Even in the case of such experimental problems, our findings reveal that at temperatures above room temperature, the higher frequency modes (with frequencies from 200 up to 600 cm^−1^) give a considerable contribution to the vibrational entropy, while on the other hand the ADPs at elevated temperatures are dominated by the low-frequency lattice vibrations. Furthermore, in the present case, the fact that the **Y** crystal has fewer vibrational modes per asymmetric unit implies that the low-frequency acoustic modes give a larger contribution to the entropy as well as to the ADPs than in the case of **W**. A mere glance at the size of the ADPs may therefore be misleading, and a thorough analysis including complementary information on the high-frequency modes is needed.

## 8. Related literature   

The following reference has been cited in the supporting information: Cliffe & Goodwin (2012 ▸).

## Supplementary Material

Crystal structure: contains datablock(s) W_100K, W_125K, W_150K, W_175K, W_200K, W_5K, W_RT, Y_100K, Y_125K, Y_150K, Y_175K, Y_200K, Y_5K, Y_RT. DOI: 10.1107/S2052252519003014/lc5101sup1.cif

GIF file: 'enhanced figure' version of Fig. 7 in the manuscript. DOI: 10.1107/S2052252519003014/lc5101sup2.gif

Structure factors: contains datablock(s) W_5K. DOI: 10.1107/S2052252519003014/lc5101W_5Ksup3.hkl

Structure factors: contains datablock(s) W_100K. DOI: 10.1107/S2052252519003014/lc5101W_100Ksup4.hkl

Structure factors: contains datablock(s) W_125K. DOI: 10.1107/S2052252519003014/lc5101W_125Ksup5.hkl

Structure factors: contains datablock(s) W_150K. DOI: 10.1107/S2052252519003014/lc5101W_150Ksup6.hkl

Structure factors: contains datablock(s) W_175K. DOI: 10.1107/S2052252519003014/lc5101W_175Ksup7.hkl

Structure factors: contains datablock(s) W_200K. DOI: 10.1107/S2052252519003014/lc5101W_200Ksup8.hkl

Structure factors: contains datablock(s) W_RT. DOI: 10.1107/S2052252519003014/lc5101W_RTsup9.hkl

Structure factors: contains datablock(s) Y_5K. DOI: 10.1107/S2052252519003014/lc5101Y_5Ksup10.hkl

Structure factors: contains datablock(s) Y_100K. DOI: 10.1107/S2052252519003014/lc5101Y_100Ksup11.hkl

Structure factors: contains datablock(s) Y_125K. DOI: 10.1107/S2052252519003014/lc5101Y_125Ksup12.hkl

Structure factors: contains datablock(s) Y_150K. DOI: 10.1107/S2052252519003014/lc5101Y_150Ksup13.hkl

Structure factors: contains datablock(s) Y_175K. DOI: 10.1107/S2052252519003014/lc5101Y_175Ksup14.hkl

Structure factors: contains datablock(s) Y_200K. DOI: 10.1107/S2052252519003014/lc5101Y_200Ksup15.hkl

Structure factors: contains datablock(s) Y_RT. DOI: 10.1107/S2052252519003014/lc5101Y_RTsup16.hkl

Supporting information file. DOI: 10.1107/S2052252519003014/lc5101sup17.cml

Supporting information file. DOI: 10.1107/S2052252519003014/lc5101sup18.mp4

Supporting figures and tables. DOI: 10.1107/S2052252519003014/lc5101sup19.pdf

CCDC references: 1899967, 1899968, 1899969, 1899970, 1899971, 1899972, 1899973, 1899974, 1899975, 1899976, 1899977, 1899978, 1899979, 1899980

## Figures and Tables

**Figure 4:**
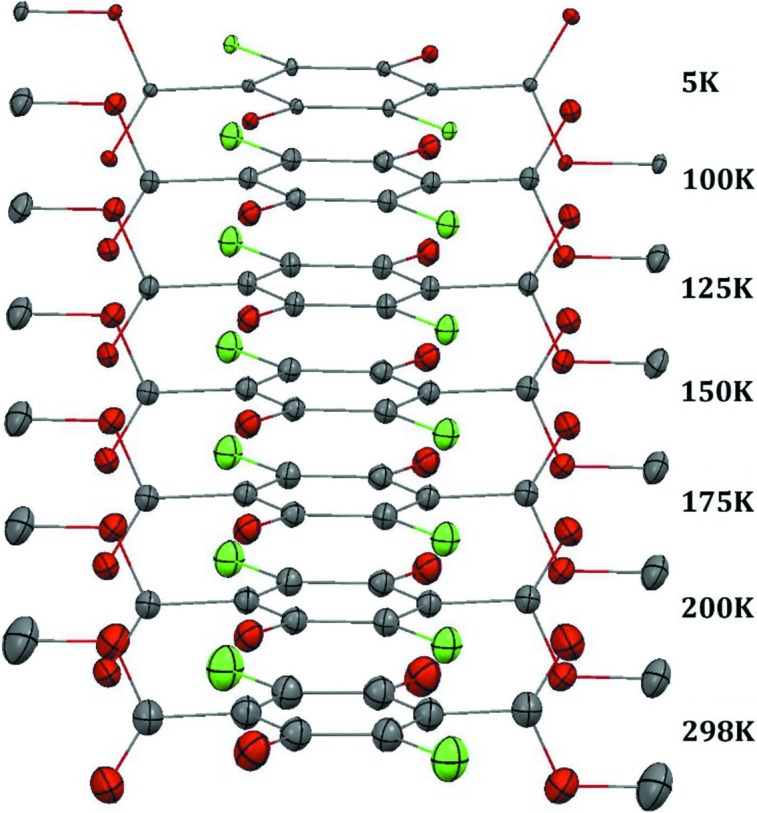
ADPs (50% probability level) at different temperatures for the **W** polymorph. Hydrogen atoms have been omitted for clarity.

**Table:** 

	**Y**	**W**
AO	*wR*, *R*	*wR*, *R*
100 K	0.179, 0.174	0.115, 0.132
125 K	0.171, 0.178	0.092, 0.111
150 K	0.184, 0.188	0.092, 0.119
175 K	0.203, 0.199	0.095, 0.119
200 K	0.199, 0.211	0.093, 0.124
Overall	0.187, 0.190	0.097, 0.121

**Table:** 

FG	*wR*, *R*	*wR*, *R*
100 K	0.121, 0.122	0.096, 0.091
125 K	0.109, 0.108	0.064, 0.086
150 K	0.116, 0.108	0.062, 0.087
175 K	0.123, 0.111	0.062, 0.084
200 K	0.126, 0.118	0.062, 0.086
Overall	0.119, 0.113	0.069, 0.087

**Table 2:** Estimates of acoustic mode frequencies from supercell calculations, from mta-NoMoRe and TLS analysis Standard uncertainties for AO and FG models are less than 1%.

	**Y**	**W**
Supercell calculations (cm^−1^)	25, 33, 37	21, 25, 28
AO model (cm^−1^)	28, 33, 33	23, 25, 30
FG model (cm^−1^)	64, 34, 35	24, 25, 30
TLS analysis† (cm^−1^)	29, 33, 35	30, 32, 37 31, 33, 37

† For **W** there are two molecules in the asymmetric unit, and TLS analysis was done separately for both of them. The frequencies listed from supercell calculations are the means over the Brillouin zone. While calculating mean values of frequencies over the Brillouin zone for acoustic modes we assume that modes are not degenerated. As both polymorphs crystallize in 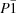, all *k*-points are equally important, thus we just calculate the sum of frequencies from all *k*-points, and then divide it by number of *k*-points.

**Table 3:** Free energy difference between **Y** and **W** polymorphs and its decomposition into contributions obtained at 300 K from different models. All values are in kJ mol^−1^

300 K	Γ	Supercell	NKA	NKA anharmonic	mta-NoMoRe_AO	mta-NoMoRe_FG
Δ*G*_total__(**Y**−**W**/2)_ (kJ mol^−1^)	4.36	−0.03	0.65	−0.65*	−1.76*	−3.04*
Δ*E*_electronic__(**Y**−**W**/2)_ (kJ mol^−1^)	−3.25	−4.30	−3.25*	−3.25*	−3.25*	−3.25*
Δ*ST*_(**Y**−**W**/2)_ (kJ mol^−1^)	−10.96	−2.99	−3.50	−2.17	−1.11	0.50
Δ*ZPE*_(**Y**−**W**/2)_ (kJ mol^−1^)	1.15	2.72	1.79	1.72	1.50	1.90
Δ*H*_vib__(**Y**−**W**/2)_ (kJ mol^−1^)	−4.49	−1.43	−1.39	−1.32	−1.12	−1.19
Estimated phase transition temp. (K)	130	300	260	380	600	2000

## Footnotes

1 It is worth noting here that since **Y** and **W** are conformational polymorphs, the differences in electronic energies between the polymorphs reflects differences arising from intermolecular interactions (lattice energies) as well as differences in intramolecular geometries (conformations). From comparison of lattice energies only, **W** seems to be the more stable form at 100 K. In **Y**, an intramolecular hydrogen bond is formed, which means that the molecules in this conformation are stabilized by 30 kJ mol^−1^ compared with molecules in the **W** conformation.

2 As from NKA we are obtaining the low-frequency acoustic and librational modes, we chose to remove frequencies corresponding to these modes from the DFT results. For **Y** four modes and for **W** eight modes were removed.
