# Supplementary figures and images for: X-ray diffraction data as a source of the vibrational free-energy contribution in polymorphic systems
Source: IUCrJ. 2019 May 8;6(Pt 4):558–71. doi: 10.1107/S2052252519003014 (PMC6608639; doi:10.1107/S2052252519003014)

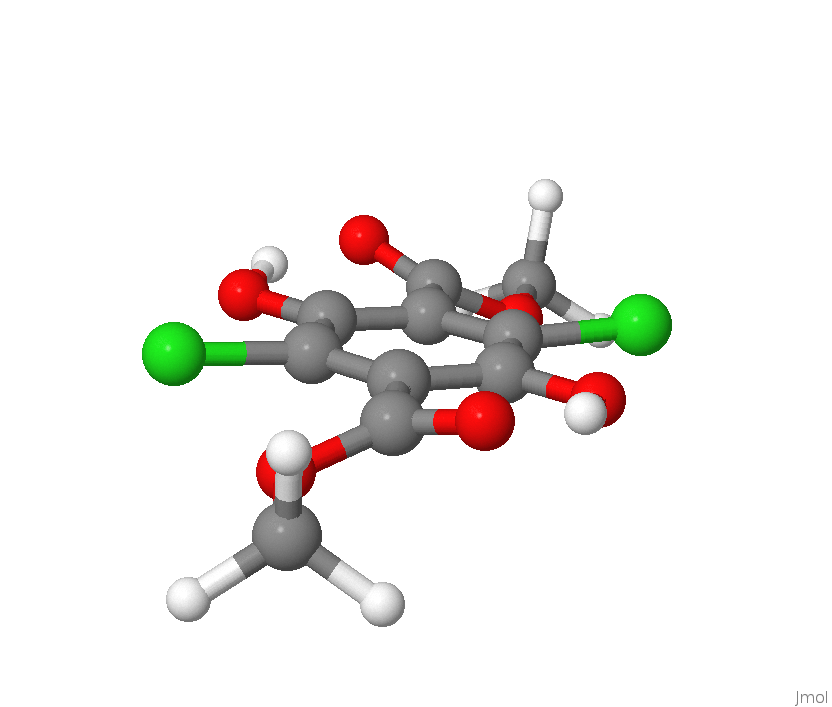

Supplement: Supplementary file 2 [file m-06-00558-sup2.gif]
