# Supplementary material for: X-ray diffraction data as a source of the vibrational free-energy contribution in polymorphic systems
Source: IUCrJ. 2019 May 8;6(Pt 4):558–71. doi: 10.1107/S2052252519003014 (PMC6608639; doi:10.1107/S2052252519003014)
Supplement: Supplementary file 19 [file m-06-00558-sup19.pdf]

IUCrJ

Volume 6 (2019)

Supporting information for article:

X-ray diffraction data as a source of the vibrational free energy contribution in polymorphic systems.

Phillip Miguel Kofoed, Anna Agnieszka Hoser, Frederik Diness, Silvia Capelli and Anders Østergaard Madsen

	Y_5K	Y_100	Y_125K	Y_150K	Y_175K	Y_200K	Y_295K
**Chemical formula**	$C_{10}H_8Cl_2O_6$	$C_{10}H_8Cl_2O_6$	$C_{10}H_8Cl_2O_6$	$C_{10}H_8Cl_2O_6$	$C_{10}H_8Cl_2O_6$	$C_{10}H_8Cl_2O_6$	$C_{10}H_8Cl_2O_6$
M_r	295.06	295.1	295.1	295.1	295.1	295.1	295.1
**Crystal system, space group**	Triclinic, *P*-1	Triclinic, *P*-1	Triclinic, *P*-1	Triclinic, *P*-1	Triclinic, *P*-1	Triclinic, *P*-1	Triclinic, *P*-1
**Temperature (K)**	5	100	125	150	175	200	295
***a*, *b*, *c* (Å)**	9.510 (1), 4.2473 (4), 7.7107 (8)	9.5239 (3), 4.2565 (1), 7.7584 (2)	9.5185 (4), 4.25574 (15), 7.7749 (3)	9.5271 (2), 4.26073 (9), 7.7960 (2)	9.5357 (3), 4.26613 (13), 7.8240 (3)	9.5488 (2), 4.27352 (11), 7.8524 (3)	9.5911 (3), 4.29992 (13), 7.9669 (3)
**α, β, γ (°)**	112.420 (5), 95.8100 (5), 106.1630 (5)	112.869 (2), 95.579 (2), 106.205 (2)	112.961 (4), 95.635 (3), 106.167 (3)	113.185 (2), 95.505 (2), 106.1658 (19)	113.317 (3), 95.508 (3), 106.125 (3)	113.547 (3), 95.379 (2), 106.125 (2)	114.358 (3), 94.954 (3), 106.143 (3)
V **(Å^3)**	268.88 (5)	270.65 (1)	270.80 (2)	271.72 (1)	273.03 (2)	274.47 (1)	279.82 (2)
Z	1	1	1	1	1	1	1
**Radiation type**	Mo $K\alpha$	Mo $K\alpha$	Mo $K\alpha$	Mo $K\alpha$	Mo $K\alpha$	Mo $K\alpha$	Mo $K\alpha$
**μ (mm^{-1})**	0.62	0.62	0.62	0.62	0.61	0.61	0.61
**Crystal size (mm)**	0.1 × 0.05 × 0.05	0.1 × 0.05 × 0.05	0.1 × 0.05 × 0.05	0.1 × 0.05 × 0.05	0.1 × 0.05 × 0.05	0.1 × 0.05 × 0.05	0.1 × 0.05 × 0.05
**Diffractometer**	Bruker *APEX*-II CCD	SuperNova, Single source at offset, Eos	SuperNova, Single source at offset, Eos	SuperNova, Single source at offset, Eos	SuperNova, Single source at offset, Eos	SuperNova, Single source at offset, Eos	SuperNova, Single source at offset, Eos
**Absorption correction**	Multi-scan *SADABS2014*/4	Multi-scan *CrysAlis PRO*,SCALE3 ABSPACK	Multi-scan *CrysAlis PRO*,SCALE3 ABSPACK	Multi-scan *CrysAlis PRO*,SCALE3 ABSPACK	Multi-scan *CrysAlis PRO*,SCALE3 ABSPACK	Multi-scan *CrysAlis PRO*,SCALE3 ABSPACK	Multi-scan *CrysAlis PRO*,SCALE3 ABSPACK
T_{min}, T_{max}	0.644, 0.748	0.726, 1.000	0.906, 1.000	0.882, 1.000	0.844, 1.000	0.833, 1.000	0.746, 1.000
**No. of measured, independent and observed [> 2.0σ(*I*)] reflections**	8746, 2614, 2389	17402, 1810, 1695	21217, 2752, 2609	21308, 2757, 2589	21405, 2756, 2545	21542, 2767, 2535	21554, 3041, 2510
R_{int}	0.039	0.037	0.023	0.024	0.025	0.026	0.028
$(\sin \theta/\lambda)_{max}$**(Å^{-1})**	0.867	0.752	0.853	0.853	0.857	0.858	0.880
$R[F^2 > 2\sigma(F^2)]$, $wR(F^2)$, S	0.037, 0.169, 3.55	0.019, 0.048, 1.28	0.017, 0.047, 1.33	0.019, 0.052, 1.43	0.022, 0.057, 1.50	0.025, 0.063, 1.60	0.050, 0.115, 2.32
**No. of reflections**	2613	1810	2752	2757	2756	2767	3041
**No. of parameters**	98	98	98	98	98	98	98
**No. of restraints**	11	11	11	11	11	11	11
$(\Delta/\sigma)_{max}$	0.083	−0.050	−0.043	−0.050	−0.055	−0.050	−0.141
$\Delta\rho_{max}$, $\Delta\rho_{min}$**(e Å^{-3})**	0.74, -1.20	0.27, −0.35	0.38, −0.29	0.44, −0.35	0.46, −0.41	0.53, −0.47	0.73, −0.55

	**W_5**	**W_100**	**W_125**	**W_150**	**W_175**	**W_200**	**W_RT**
**Chemical formula**	$C_{10}H_8Cl_2O_6$	$C_{10}H_8Cl_2O_6$	$C_{10}H_8Cl_2O_6$	$C_{10}H_8Cl_2O_6$	$C_{10}H_8Cl_2O_6$	$C_{10}H_8Cl_2O_6$	$C_{10}H_8Cl_2O_6$
M_r	295.08	295.08	295.08	295.08	295.08	295.08	295.08
**Crystal system, space group**	Triclinic, *P*-1	Triclinic, *P*-1	Triclinic, *P*-1	Triclinic, *P*-1	Triclinic, *P*-1	Triclinic, *P*-1	Triclinic, *P*-1
**Temperature (K)**	5	100	125	150	175	200	294
***a*, *b*, *c* (Å)**	9.8021 (4), 7.7322 (4), 10.4934 (5)	9.8043 (4), 7.7484 (4), 10.5027 (5)	9.8090 (4), 7.7577 (3), 10.5083 (5)	9.8127 (2), 7.76700 (16), 10.5170 (3)	9.8178 (2), 7.77841 (17), 10.5270 (3)	9.8231 (2), 7.79072 (17), 10.5376 (3)	9.8461 (3), 7.84791 (19), 10.5783 (3)
**α, β, γ (°)**	116.848 (4), 124.3970 (4), 88.7890 (3)	116.731 (4), 124.349 (4), 88.853 (3)	116.688 (5), 124.329 (5), 88.869 (4)	116.638 (2), 124.323 (3), 88.869 (2)	116.595 (2), 124.299 (3), 88.884 (2)	116.557 (2), 124.277 (3), 88.896 (2)	116.412 (3), 124.159 (3), 88.976 (2)
***V* (Å^3)**	555.15 (1)	557.98 (1)	559.65 (4)	561.46 (2)	563.58 (2)	565.79 (2)	575.36 (3)
Z	2	2	2.0	2.0	2.0	2.0	2
**Radiation type**	Mo *K*α	Mo *K*α	Mo *K*α	Mo *K*α	Mo *K*α	Mo *K*α	Mo *K*α
**μ (mm^{-1})**	0.60	0.60	0.60	0.60	0.59	0.59	0.58
**Crystal size (mm)**	2.0 × 0.5 × 0.35	2.0 × 0.5 × 0.35	2.0 × 0.5 × 0.35	2.0 × 0.5 × 0.35	2.0 × 0.5 × 0.35	2.0 × 0.5 × 0.35	2.0 × 0.5 × 0.35
**Diffractometer**	Bruker *SMARTAPEX2* area detector	SuperNova, Single source at offset, Eos	SuperNova, Single source at offset, Eos	SuperNova, Single source at offset, Eos	SuperNova, Single source at offset, Eos	SuperNova, Single source at offset, Eos	SuperNova, Single source at offset, Eos
**Absorption correction**	Multi-scan *SADABS2014*/4	Multi-scan *CrysAlis PRO*, SCALE3 ABSPACK	Multi-scan *CrysAlis PRO*, SCALE3 ABSPACK	Multi-scan *CrysAlis PRO*, SCALE3 ABSPACK	Multi-scan *CrysAlis PRO*, SCALE3 ABSPACK	Multi-scan *CrysAlis PRO*, SCALE3 ABSPACK	Multi-scan *CrysAlis PRO*, SCALE3 ABSPACK
T_{min}, T_{max}	0.647, 0.748	0.473, 1.000	0.549, 1.000	0.534, 1.000	0.586, 1.000	0.586, 1.000	0.574, 1.000
**No. of measured, independent and observed [> 2.0σ(*I*)] reflections**	24786, 7048, 6026	96497, 5428, 4931	20138, 2810, 2564	65857, 5858, 5141	66140, 5867, 5015	66436, 5848, 4866	62436, 6432, 4511
R_{int}	0.039	0.050	0.039	0.041	0.042	0.042	0.049
**(sin θ/λ)$_{max}$(Å^{-1})**	0.927	0.856	0.691	0.880	0.882	0.880	0.880
$R[F^2 > 2\sigma(F^2)]$, $wR(F^2)$, S	0.027, 0.077, 1.47	0.023, 0.055, 1.30	0.023, 0.051, 1.18	0.025, 0.057, 1.26	0.027, 0.059, 1.26	0.028, 0.062, 1.29	0.037, 0.080, 1.35
**No. of reflections**	7048	5428	2810	5858	5867	5848	6432
**No. of parameters**	195	195	195	195	195	195	195
**No. of restraints**	16	16	16	16	16	16	16
$\Delta\rho_{max}$, $\Delta\rho_{min}$(e Å^{-3})	0.68, −0.93	0.45, −0.37	0.26, −0.38	0.34, −0.49	0.36, −0.42	0.36, −0.41	0.42, −0.48

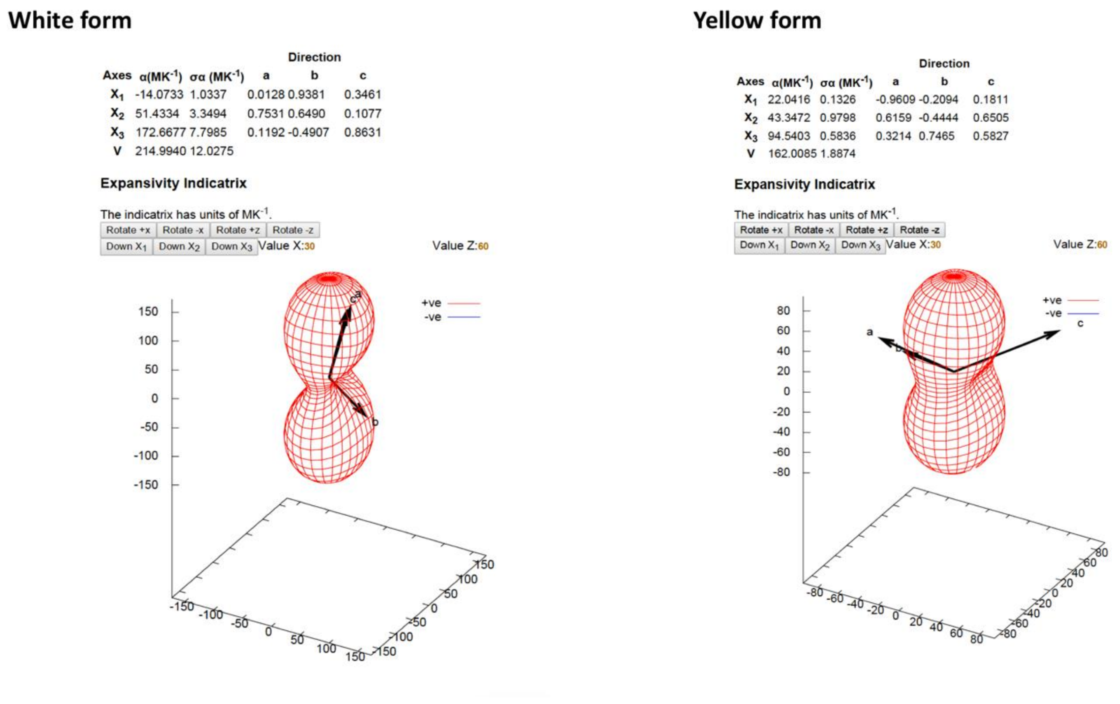

Figure S1. Expansivity indicatrices calculated by the PASCal software. (Cliffe & Goodwin, 2012) Left: White form. Right: Yellow form.

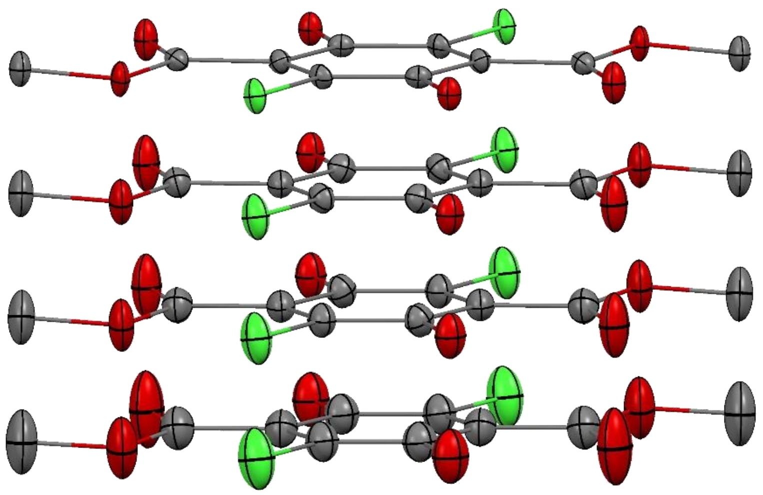

Figure S2. ADPs obtained by Dunitz and co-workers for Y polymorph

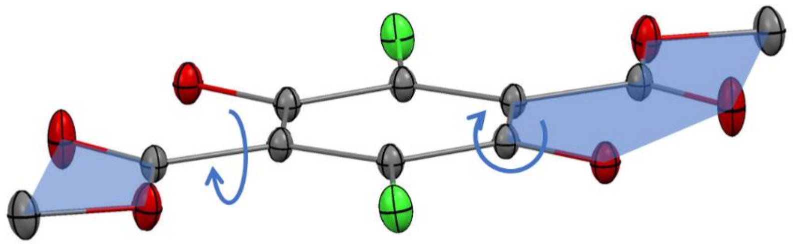

(a)

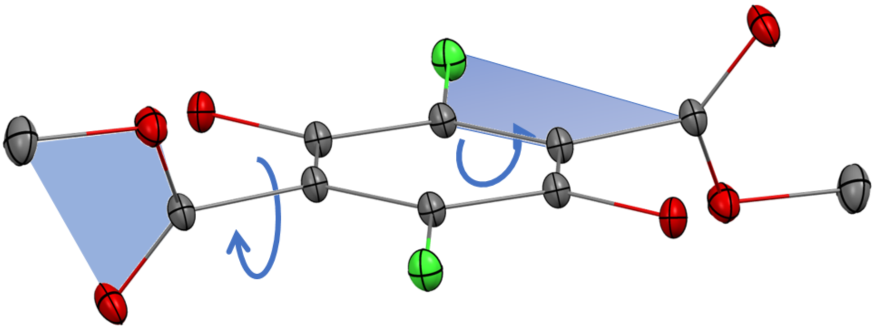

(b)

Figure S3. Description of internal vibrational modes in NKA models for yellow (a) and white (b).

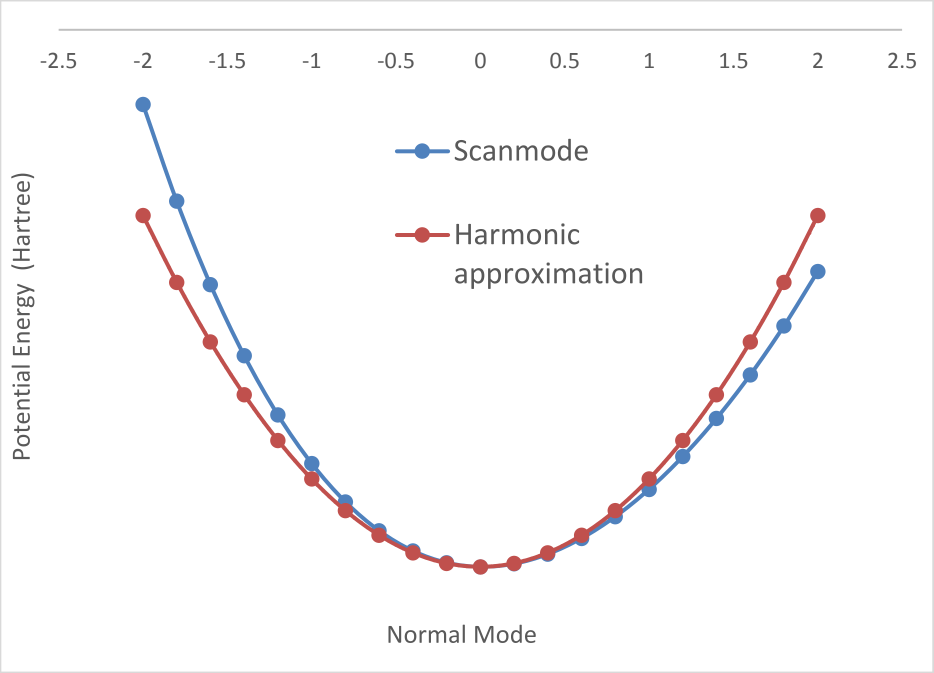

Figure S4. Energy scan along normal mode vector for Y, mode number 5.

Figure S5. PEANUT plots – difference between models from *NoMoRe* and from routine X-ray refinements (*Scaling applied in PEANUT 3.08*).

**Y**	**100K**	**150K**	**200K**
**AO**			
**FG**			

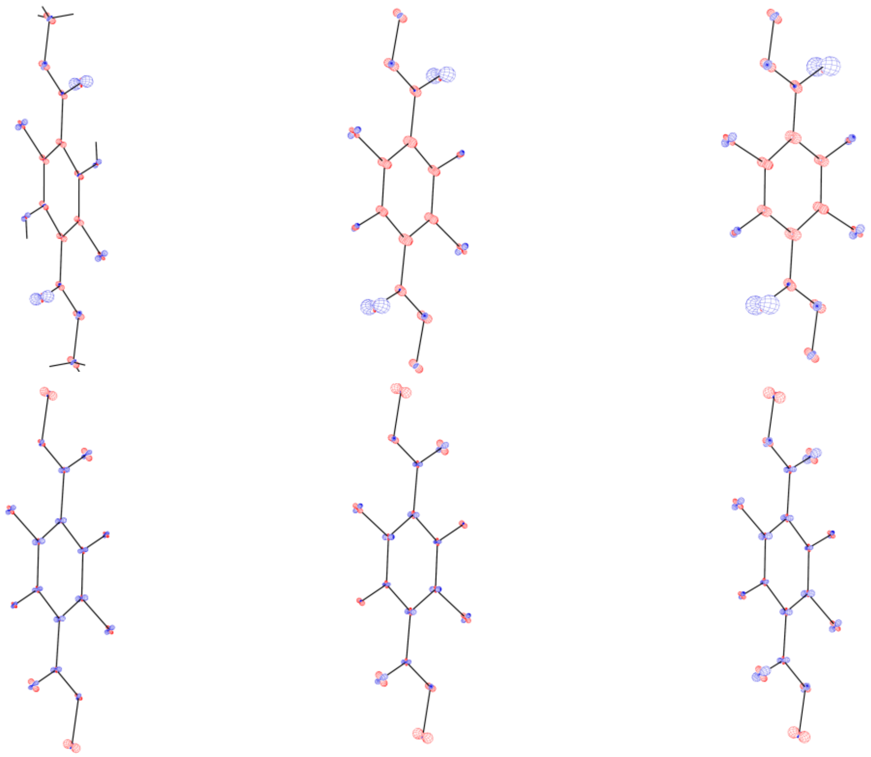

**W_I**	**100K**	**150K**	**200K**

AO

FG

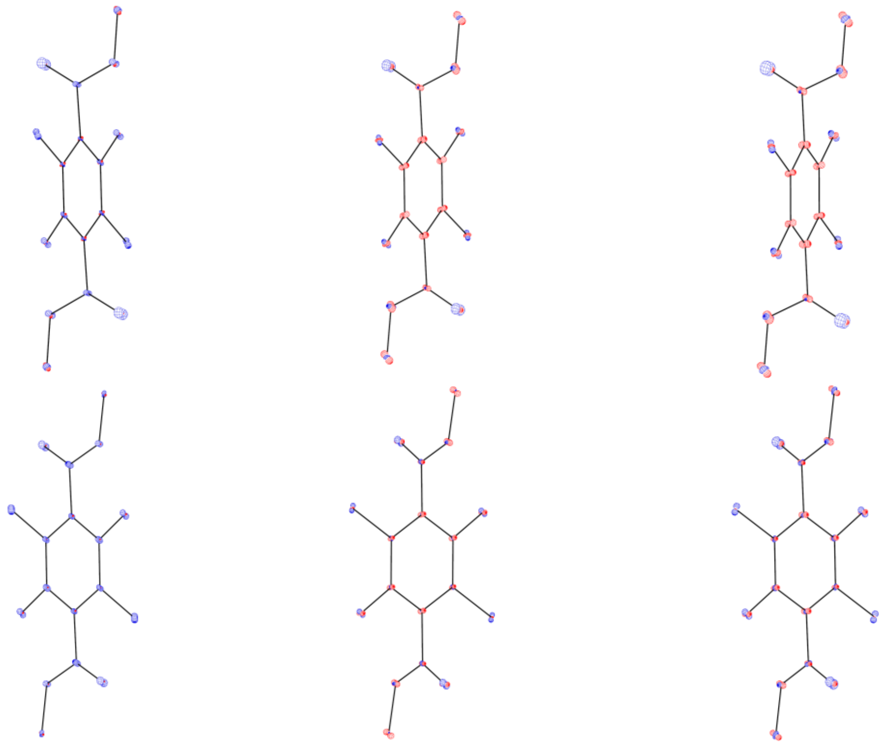

W_II **100K** **150K** **200K**

AO

FG

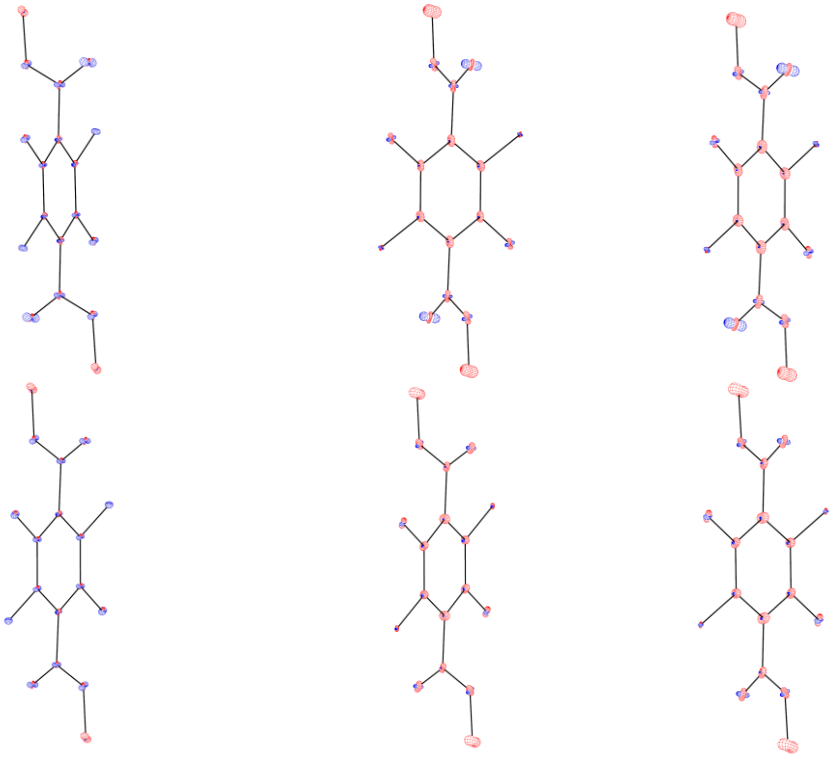

Table S2. Final statistics and frequencies obtained for yellow and white after NKA refinement

Polymorph	Model	Statistics after refinement: Observations/Restrains/Parametrs R2ij	Frequencies (cm^{-1})	Gruneisen parameters	Main Eigenvector components
**Yellow**	TLS+ gruneisen+ internal_modes (see Fig S2a)	756 / 39 /82 R2ij: 0.084	35 (1)	7.0(3)	54% Lx + 46% Ly
			48 (1)		95% Lz
			76 (7)		45% Lx + 50% Ly + 5% Lz
			47 (2)	5.0(8)	Tx
			49 (2)	6.2(8)	Ty
			37 (1)	5.0(2)	**50% Tz + 50% U1**
**White Molecule 1**	TLS+ gruneisen+ internal_modes (see Fig S2b)	756 / 39 / 80 R2ij: 0.078	36(1)		27% Lx + 73% Ly
			53(2)	7.1(6)	5% Lx + 92% Lz
			79(7)		68% Lx + 25% Ly + 8% Lz
			35(1)		85% Tx + 13% Ty
			33(1)		14% Tx + 84% Ty
			48(2)	4.7(8)	**63% Tz + 16% U1 + 20% U2**
**White Molecule 2**	TLS+ gruneisen+ internal_modes (see Fig S2b)	756 /39 / 81 R2ij: 0.076	65(2)		35% Lx + 32% Ly + 32% Lz
			58(2)	5.1(8)	37% Lx + 61% Lz
			35(1)		28% Lx + 65% Ly
			36(1)		83% Tx + 5% Ty + 9% Tz **+ 3% U1**
			32(1)		6% Tx + 93% Ty
			51(2)	5.7(6)	**9% Tx + 71% Tz + 14% U2 + 3% U1**

U1 = Butterfly motion; U2 = COO group rotation

Table S3. Energies computed for Y and W from supercell and Γ point DFT computations.

300K	E_Y Supercell	E_Y Γ	E_W supercell	E_W Γ
E_{total} /kJ·mol^{-1}	-4611791	-4611779	-9223583	-9223566
$E_{electronic}$/kJ·mol^{-1}	-4612228	-4612213	-9224447	-9224419
ST/kJ·mol^{-1}	85,8	77,5	177,6	176,9
ZPE/kJ·mol^{-1}	476,5	470,3	947,5	938,2
H_{vib}/kJ·mol^{-1}	45,3	41,4	93,5	91,9

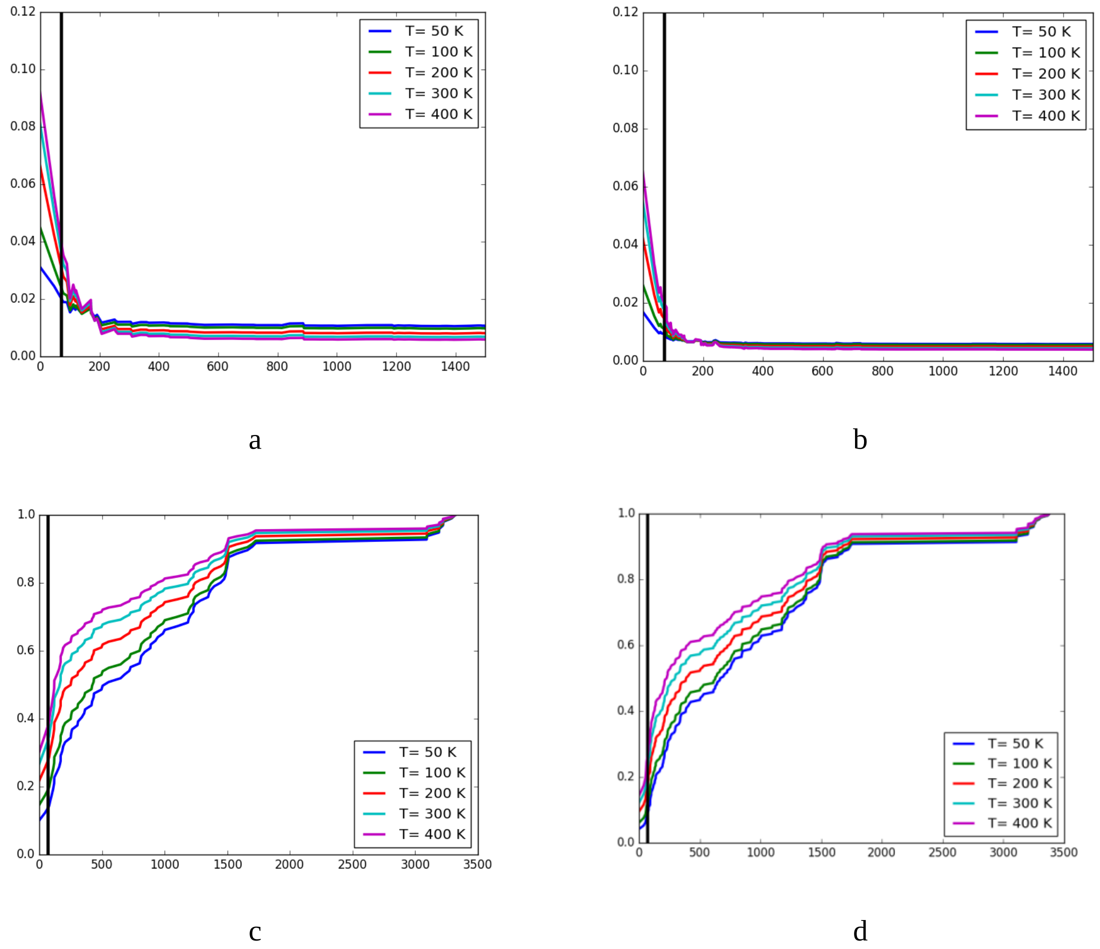

Figure S6. Contribution of given frequency to Uiso for **Y** and **W (a)** and **(b),** cumulative contribution of frequencies to Uiso for **Y** and **W (c)** and **(d).**

References

Cliffe, M. J. & Goodwin, A. L. (2012). *J. Appl. Crystallogr.* **45**, 1321–1329.
